# Adult film ratings to stop kids lighting up

**DOI:** 10.2471/BLT.16.020216

**Published:** 2016-02-01

## Abstract

A growing body of evidence showing that on-screen smoking makes more kids light up is increasing pressure to assign adult ratings to films that show smoking. Gary Humphreys reports.

Sanghamitra Pati’s memories of Indian film are veiled in tobacco smoke.

“First, there was so much smoking on screen,” says the resident of Bhubaneswar, capital of the Indian state of Odisha.

“The hero would light a cigarette to express his frustration, or his masculinity. He would light a cigarette to think!

“And then there was the smoke in the cinema itself. It was all normal, of course. All accepted.”

These days it is not so accepted, according to Pati, a researcher with the Public Health Foundation of India.

“Heroes still smoke in Bollywood films, but less than they used to, and each time there is a smoking scene, a tobacco control message comes up on the screen,” she says.

“Heroes still smoke in Bollywood films, but less than they used to.”

Sanghamitra Pati

Several factors have driven the change in attitudes to on-screen smoking in India in the past decade or so, but among the most important is the emergence of evidence showing that smoking in films makes people, especially young people, smoke their first cigarette.

That evidence continues to grow and in the past few years has yielded several compelling studies linking exposure to on-screen smoking with youth smoking initiation.

For example, a 2011 study of more than 5000 adolescents in the United Kingdom of Great Britain and Northern Ireland found that 15-year-olds who saw the most films with smoking imagery were 73% more likely to have tried smoking than those who had seen the fewest.

Research in Canada, Norway and the United States of America had similar results.

“Studies show that 37% of all new young smokers in the United States (US) start smoking as a result of their exposure to on-screen smoking,” says tobacco control advocate Professor Stanton Glantz, who leads the Smoke Free Movies initiative at the University of California, San Francisco.

“That’s a bigger effect than conventional cigarette advertising,” says Glantz.

**Figure:**
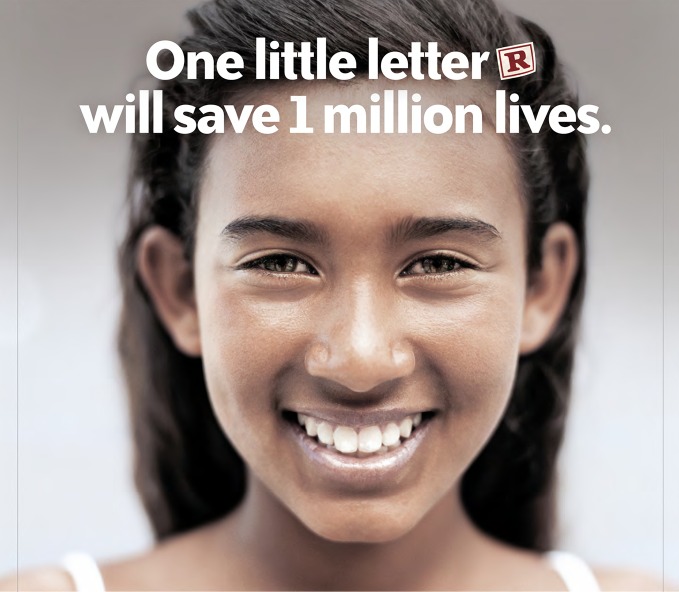
Poster for the Smoke Free Movies initiative

Courtesy of the Smoke Free Movies initiative

Since 2005, countries that are parties to the World Health Organization (WHO) Framework Convention on Tobacco Control have been required to adopt strict tobacco control measures, including advertising and marketing bans.

As a result, traditional outlets for tobacco advertising, such as in magazines and television, have been reduced and the number of occurrences of smoking or other tobacco use in film has increased in many countries, according to a new WHO report *Smoke-free movies: from evidence to action* (third edition) released this month.

This trend was observed in India, after tobacco advertising in other media was prohibited, and in many other parts of the world.

“There’s a fairly clear pattern that when tobacco advertising and promotion is restricted in one medium, it will show up in another,” Glantz says.

That it should show up in film is perhaps not surprising, given the tobacco industry’s long history of using showbiz and the film industry to push its products.

Glantz and other health advocates believe that one way to reduce children’s exposure to on-screen smoking is to assign an adult rating to films depicting smoking.

The idea is simple: adult-rated films make less money, so film studios will leave the smoking out of movies that they want to sell to children. As a result, children will see less smoking in films and fewer of them will take up the highly addictive habit.

**Figure:**
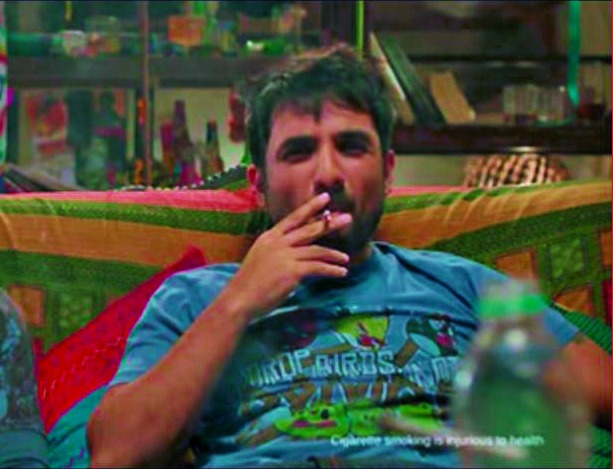
Screenshot from Bollywood film *Go Goa Gone* released in 2013

Courtesy of Sanghamitra Pati

At least half of all smokers die from smoking-related diseases, such as lung cancer, roughly six million people each year. But so far no country has used youth ratings – G (general audiences), PG (parental guidance suggested) and PG-13 (parents strongly cautioned) – to protect young people from the harms of smoking, even countries that have taken a bold stance on smoking in films, such as India.

Why? Two arguments are often made.

First, imposing a rating restricts freedom of expression. The defence-of-creativity argument has often been used to resist tobacco control measures. In India, a group of Bollywood producers recently said that the requirement to show a tobacco control message during films was “killing creativity”.

The defence-of-creativity argument is also the basis of exception clauses in policy documents drawn up by five of the six major Hollywood studios to keep smoking imagery out of films made for children.

However, as Glantz points out, many things are already excluded from youth-rated films under current rating systems, including frontal nudity and bad language.

“So the creativity argument doesn’t really hold up,” Glantz says, adding that there is nothing to stop artists creating films with smoking in them. These films should not be shown to children.

The second argument is that ratings agencies reflect the public view of what is acceptable.

This argument was used by the British Board of Film Classification (BBFC), the United Kingdom’s board of censors, in response to the above-cited United Kingdom study, thus rejecting calls for films with smoking scenes to be rated like films showing sex and violence.

In a letter to the authors of the 2011 United Kingdom study, the BBFC, which is funded by fees it charges the film industry for classifying films, concluded that its current guidelines take “due account of the available evidence of harm; and reflect the clear wishes of the public”.

The Motion Picture Association of America (MPAA), which represents the interests of six major US film studios, takes a similar line.

“It is important to remember that we rate for a majority of American parents. And surveys indicate we are on track,” says MPAA spokesperson Howard Gantman.

“Smoking is not high on the list of concerns of the majority of parents as they feel doctors, schools and parents are playing the necessary part in education,” Gantman says, citing a survey commissioned by the Classification and Rating Agency that rates films for the MPAA.

The 2015 survey found that 39% of the 1488 parents surveyed were “very concerned” with smoking content in films, compared with 80% of respondents who were very concerned about graphic sex scenes, hard drugs use (70%) and graphic violence (64%).

Gantman argues that most films with smoking scenes are rated for people aged over 17 or 18 years of age.

“Our statistics show that 53% of the 6174 films that were rated between May 2007 and May 2015 contained one incidence or more of smoking. Of the movies that contained smoking: 73% were rated R (no one under 17 admitted without an accompanying parent or guardian) or NC-17 (no one under 18 admitted) and 21% were rated PG-13 while 6% were rated PG, Gantman says, referring to MPAA’s internal statistics.

Glantz, however, dismisses internal statistics and surveys commissioned for the film industry as biased. “Mississippi State University researchers analysing a scientifically valid national sample of US adults in 2006 found that 70% called for R-ratings in movies that show smoking, unless the film clearly demonstrates the dangers of smoking or is necessary to represent smoking of a real historical figure,” he says.

Film industry arguments aside, the major film studios have been at pains to improve their image on smoking in their youth-rated film content by adopting polices to discourage or – as was the case of Disney last year – to put an end to smoking in their youth-rated films.

These initiatives suggest that the film companies themselves recognize that many American parents may not be comfortable with on-screen smoking.

While smoking imagery in the major US studios’ films has dropped in recent years, it has increased in films made elsewhere, including in the independent sector.

Youth-rated movies produced by four major studios – Disney, Paramount, Universal and Warner Brothers – were 100% smoke free during the first half of 2015.

But by December 2015, every US studio, including multimedia giants Fox and Sony as well as independent film companies, such as Lionsgate and Weinstein, had released youth-rated films with smoking and the total number of such films was about the same as in each of the previous four years.

Although Sony published its corporate tobacco guidelines in 2012 pledging to reduce smoking in its films, 60% of its PG-13 films last year still included smoking scenes, according to data from Breathe California’s Thumbs Up! Thumbs Down! project analysed by the University of California, San Francisco.

But does assigning youth ratings to films with smoking really make a difference?

After all, many children now view film on DVD or live stream or download content from the Internet, and do not even need to go to a cinema.

“With the growth of piracy simply putting an R-rating on a movie that young people want to see will simply fuel illegal viewing,” argues Gantman.

Dr Armando Peruga, programme manager of WHO’s Tobacco Free Initiative, agrees: “Keeping smoking out of youth-rated films is one of the most powerful ways to protect children from the harms of smoking.”

“Children may be exposed to adult-rated content through the Internet, but in many parts of the world, traditional film and television distribution which uses the rating system is still the norm,” Peruga says.

Companies should be required to declare any benefits received from the tobacco industry and to remove identifiable tobacco brands from their films, Peruga argues. In addition, film theatres should be required to show health warnings about smoking before and after films.

For Glantz, the idea of assigning films with smoking imagery an adult rating is not to stop youth from seeing films, but to create an economic incentive for producers to leave smoking out of the films that kids see the most.

“US films are seen all over the world, so modernizing the rating system to get smoking out of youth-rated films in the US will protect youth all over the world,” Glantz says.

“US films are seen all over the world, so modernizing the rating system to get smoking out of youth-rated films in the US will protect youth all over the world.”

Stanton Glantz

